# Prevalence of Rotavirus in Diarrheic Piglets on RVA-Vaccinated and Non-Vaccinated Farms

**DOI:** 10.3390/pathogens14101055

**Published:** 2025-10-18

**Authors:** Weronika Rybkowska, Aleksandra Woźniak, Nicole Bakkegård Goecke, Lars Erik Larsen, Piotr Cybulski, Tomasz Stadejek

**Affiliations:** 1 Department of Pathology and Veterinary Diagnostic, Institute of Veterinary Medicine, Warsaw University of Life Sciences-SGGW, Nowoursynowska 159C, 02-776 Warsaw, Poland; tomasz_stadejek@sggw.edu.pl; 2 Department of Veterinary and Animal Sciences, University of Copenhagen, 1870 Frederiksberg, Denmark; nbgo@sund.ku.dk (N.B.G.); lael@sund.ku.dk (L.E.L.); 3 Goodvalley Agro S.A., Dworcowa 25, 77-320 Przechlewo, Poland; piotr.cybulski@goodvalley.com

**Keywords:** rotavirus, piglet diarrhea, vaccination, real-time PCR

## Abstract

Rotaviruses (RVs) are an important cause of piglet diarrhea. This study aimed to determine the prevalence of rotavirus A, B, and C (RVA, RVB and RVC) in two RVA-vaccinated (VAC) and four non-vaccinated (NON-VAC) farms, and the impact of RVA vaccination on production parameters. Additionally, RVs prevalence in consecutive weekly groups from one vaccinated and one non-vaccinated farm was assessed. Diarrheic feces or ileum content were screened for RVs using real-time RT-PCR. In VAC, no RVA or RVB was detected, while RVC was found in all the samples (15/15). In NON-VAC, RVA, RVB, and RVC were detected in 10.5%, 13.2%, and 52.6% of samples, respectively. RVC was the most prevalent species in longitudinal study, while RVA was found in single samples. RVB was detected in one sample from the vaccinated farm, and in four out of five groups from the non-vaccinated farm. The pre-wean mortality and weaning weight were lower in the vaccinated than in the non-vaccinated farm. Low RVA prevalence and no noticeable improvement in weaning outcomes suggest vaccination was probably unjustified. Our study emphasizes the importance of comprehensive screening before and after vaccination and highlights the importance of including RVB and RVC in diagnostics of neonatal diarrhea.

## 1. Introduction

Rotaviruses (RVs) belong to the genus *Rotavirus* in the family *Sedoreoviridae* and are non-enveloped, wheel-shaped viruses with a genome composed of 11 double-stranded RNA (dsRNA) segments (assigned 1–11), each coding for at least one viral protein: six structural (VP1-VP4, VP6, VP7) and five or six non-structural ones (NSP1-NSP5/6) [1,2,3]. Based on the sequence and antigenic differences in VP6, the genus is classified into nine species (RVA-RVD and RVF-RVJ) and two putative ones (RVK, RVL) [1,2,3]. Rotavirus replicates in enterocytes, mainly in the jejunum and ileum, generating cell exfoliation and villi atrophy, what leads to malabsorption of solutes and osmotic influx in the intestinal lumen [4,5]. Rotaviral infections are an important cause of severe, even fatal, diarrheal illness in both children and young animals [6].

Suckling piglet diarrhea is one of the most common issues in the modern swine husbandry, affecting both pork production profitability and animal welfare. Diarrhea might rapidly cause dehydration, weight loss, or even increased mortality [7]. In Europe, the most economically important viral enteropathogens are RVs as the prevalence of porcine epidemic diarrhea virus (PEDV) and transmissible gastroenteritis coronavirus (TGEV) is low [8,9,10,11].

To date, five species of RVs have been detected in swine (RVA, RVB, RVC, RVE, RVH) [5]. Multiple RV species are known to co-circulating in swine populations [12]. RVE was detected only once in 1986 and has not been studied genetically; therefore, it has been excluded from the current RVs classification [13]. Historically, RVA has been considered the most prevalent and pathogenic member of the genus *Rotavirus* in pigs worldwide; however, recently RVC has been identified as an important cause of porcine neonatal diarrhea [14,15,16]. In Europe, high RVC detection rates have been reported in diarrheic suckling piglets [8,17,18]. Interestingly, there was a variation in the detection rates of RVB reported from cases of suckling piglet diarrhea in Europe. RVB infections are more common in older animals (over 55 days old) [14]. However, there are studies indicating that RVB is widespread in all age groups [12,19]. Cases of RVB outbreaks in suckling piglets, characterized by high morbidity and mortality, have been reported [20,21,22].

High prevalence of RV infections in swine indicates the need for effective prevention measures. However, control of RV spread poses difficulties since RVs are highly resistant to environmental conditions, making effective disinfection difficult [5]. The severity of diarrhea and its impact on production parameters, as well as occurrence of pathogens, may depend on internal biosecurity and hygiene. Rigorous biosecurity measures can reduce the number and the severity of diarrhea episodes among suckling piglets [23]. On the other hand, high standards of hygiene limit the exposure of sows, and could also compromise the level of maternal immunity transferred to piglets. Natural planned exposure (NPE) is a control strategy based on sows’ exposure to the circulating viruses. NPE has the potential to control the disease caused by RVA as well as non-cultivable RVs species, such as RVC or RVB. Nevertheless, this method poses the risk of unintended spread of other pathogens [24]. The control strategies of rotavirus diarrhea in piglets include also dam vaccination with commercial or autogenous vaccines against RVA. The effectiveness of RVA vaccinations in pigs, against RVA genotypes not included in the vaccine composition, is unknown [25]. Certainly, there is no cross-protection against other rotavirus species [15]. However, the RVB and RVC are difficult to isolate so autogenous vaccines are not an option [15].

There are reports indicating that immunization against RVA, increases the prevalence of RVB and RVC in the swine population [21,26]. Moreover, it has been observed that high RVA shedding is associated with increased odds of low shedding of RVB or RVC [24]. Therefore, the efficacy of immunization against RV induced diarrhea may be ineffective and economically questionable, as RVA may be, to some extent, replaced by other RV species. Thus, the aim of the study was to determine the prevalence/occurrence of RVA, RVB and RVC in RVA-vaccinated and non-vaccinated farms and to evaluate the impact of RVA vaccination on several production parameters to assess the efficiency of vaccination against RVA.

## 2. Materials and Methods

### 2.1. Farms Description

Six high-performing porcine reproductive and respiratory syndrome virus (PRRSV)-negative sow farms (S1–S6), with populations ranging from 1400 to 5200 sows in each, were selected for this study. The production system on each farm is based on weekly farrowing groups, and all the animals undergo similar husbandry and veterinary practices. The farms belong to one of the largest pork producers in Poland and maintain genetic uniformity. All the animals were reared on a slatted floor following an all-in all-out system under conditions complying with the legal welfare requirements of Council Directive 2008/120/EC of 18 December 2008 laying down minimum standards for the protection of pigs.

All farms had a history of neonatal diarrhea characterized by high morbidity (up to 100%) and variable mortality among affected litters. The farms were regularly tested for enteric pathogens, and TGEV and PEDV infections had not occurred in the last five years. The enteropathogens detected in routine health monitoring included pathogenic *Escherichia coli*, anaerobic bacteria, RVA, and RVC. The presence of other RV species (RVB and RVH) was not evaluated.

Vaccination against RVA has been conducted on farm S1 and S2 (VAC farms) since 2019. Nevertheless, the efficiency of the immunization program was not always satisfactory and the antibiotic treatment of piglets with diarrhea was necessary. However, its efficacy was also limited. Autogenous RVA vaccine was administered to gilts and sows three weeks prior to the expected farrowing. For gilts, one more dose was given six weeks before farrowing. Every 2 mL dose of the product contains inactivated virus (RP ≥ 1) and 0.52 mL of the commercial oil adjuvant Montanide ISA 35 VG (Seppic SA, La Garenne Colombes, France). The strain (G5P [7]) included in the vaccine was isolated from farm S1 and S2 in 2019.

On farm S3–S6 (NON-VAC farms) no immunization against RVA has been implemented. In all farms, sows and gilts were immunized with the vaccines Suiseng Coli/C and Suiseng Diff/A (Laboratorios Hipra S.A., Amer, Spain) against pathogenic *E. coli*, *Clostridium perfringens* type A and C, *Clostridium novyi* type B, and *Clostridioides difficile*. The vaccines were administered according to the manufacturer’s recommendations.

### 2.2. Sample Collection, Processing and Real-Time RT-PCR

#### 2.2.1. Initial Screening for RVA, RVB and RVC

**Figure 1:**
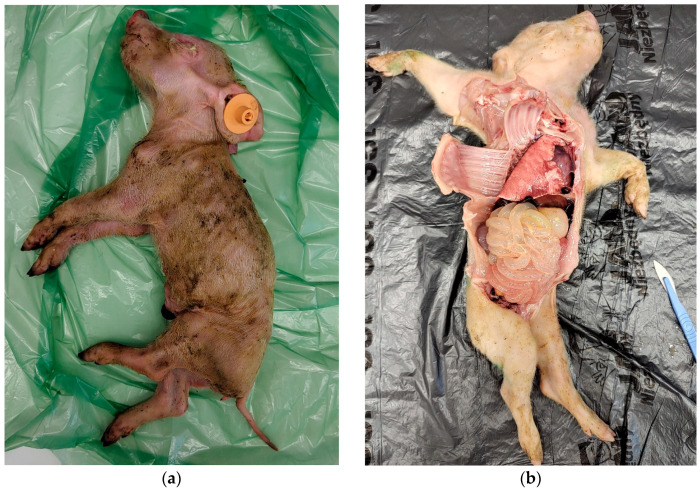
Post-mortem appearance of piglets with typical findings for rotavirus infections, including visible signs of malnutrition (**a**) and thinning of the intestinal wall (**b**).

The samples from all six farms (S1–S6) were obtained as a part of routine diagnostic or monitoring protocols, so the approval of the local ethics committee was not required. Intestinal samples (ileum and colon content) were collected by a veterinarian from dead piglets (Figure 1), up to three weeks old, showing signs of dehydration and malnutrition, and the samples were tested individually. This study was based on diagnostic submissions and as a result, the number of visits per farm (one to three) and the number of samples collected per visit (one to seven) varied, and sampling was performed across different seasons and years. The number of samples and collection dates are shown in Table 1. All samples were transported to the laboratory of the Department of Pathology and Veterinary Diagnostics of the Faculty of Veterinary Medicine at the Warsaw University of Life Sciences under refrigerated conditions. RNA was extracted using the Kylt RNA/DNA Purification kit (SAN Group Biotech Germany GmbH, Höltinghausen, Germany). The extraction was conducted in accordance with the manufacturer’s instructions. Extracted RNA was screened for RVA, RVB, RVC and RVH using primer and probe sequences described elsewhere [19]. For the real-time RT-PCR analyses either SensiFAST Probe No-ROX One Step Kit (Bioline, London, UK) or virotype Mix+IC (TAMRA)-RNA kit (Indical Bioscience, Leipzig, Germany) were used depending on availability of the products. A sample with a cycle threshold (Ct) ≤ 37 in the real-time PCR assays was considered positive.

Additionally, the presence of genetic material from porcine coronaviruses (PEDV, TGEV and swine delta coronavirus (SDCoV)) was assessed using the commercial real-time RT-PCR kit VetMAX PEDV/TGEV/SDCoV Kit (Thermo Fisher Scientific, Waltham, MA, USA). The reactions were conducted in accordance with the manufacturers’ instructions.

#### 2.2.2. Longitudinal Testing in Two Farms

The farm management decided on conducting longitudinal study on two randomly chosen farms (S2 and S6) (one VAC and one NON-VAC) to gain better insight into the farms’ health status. Therefore, the approval of the local ethics committee was not required.

To assess the prevalence of RVs in five consecutive weekly groups, the samples from farm S2-VAC and S6-NON-VAC were collected between 4 July 2023 and 1 August 2023. Each week, up to five farrowing pens showing clinical signs of diarrhea were randomly selected. Within each selected pen, rectal swabs were collected from two random diarrheic piglets. Moreover, up to eight intestinal samples were collected from emaciated dead piglets per week. All samples were collected by veterinarian from piglets up to seven days old. Overall, samples were collected from 6 to 18 animals per week group. The number of both sample types collected in each week are presented in Table 2. Intestinal samples (ileum content) were tested individually. Prior to RNA extraction, fecal samples from each pen were pooled with two samples per pool. RNA was extracted using IndiSpin QIAcube HT Pathogen Kit (Indical Bioscience, Leipzig, Germany) and the extraction robot Qiacube HT (Qiagen, Hilden, Germany). The extraction was conducted in accordance with the manufacturer’s instructions. The samples were analyzed for the presence of RVA, RVB and RVC using the Ag-Path-ID One-Step Master Mix (Thermo Fisher Scientific, Waltham, MA, USA) and primer and probe sequences described in [19]. A sample with a Ct ≤ 37 in the real-time RT-PCR assays was considered positive.

The presence of genetic material from porcine coronaviruses (PEDV, TGEV, and SDCoV) was assessed using the previously described method.

### 2.3. Biosecurity Scoring

To evaluate potential risk factors for spread of RV and to exclude the possibility that differences in biosecurity measures could have influenced the RV prevalence in the analyzed farms, both external and internal biosecurity levels were assessed using the ‘Biocheck.UGent’ questionnaire (https://biocheckgent.com/en/questionnaires/pigs-indoor; accessed on 6 December 2023), with special focus on internal biosecurity, particularly in the farrowing units. The questionnaire includes 109 questions divided into subcategories covering external and internal biosecurity. Internal biosecurity subcategories are as follows: ‘Disease management‘; ‘Farrowing and suckling period’; ‘Nursery unit’; ‘Finishing unit’; ‘Measures between compartments, working lines and use of equipment’; ‘Cleaning and disinfection’. Category scores range from 0% (worst-case scenario) to 100% (best-case scenario).

### 2.4. Key Performance Indicators Assesment

The data regarding key performance indicators (number of weaned piglets per litter, average weaning weight, and pre-weaning mortality rate) were obtained from a commercial management system used at the study farms (Cloudfarms; Cloudfarms AS, Bratislava, Slovakia).

### 2.5. Statistical Analysis

Statistical analyses were performed using GraphPad Prism version 8 for Windows (GraphPad Software, San Diego, CA, USA). The prevalence of RV species in the different farms was compared using Fischer’s exact test. Comparison of production parameters in S2-VAC and S6-NON-VAC farms was performed using Mann–Whitney test. This test was also used to compare Ct values in coinfected and non-coinfected pigs. A *p*-value < 0.05 was set as the statistically significant level.

## 3. Results

### 3.1. Detection of RVA, RVB and RVC with Real-Time RT-PCR

#### 3.1.1. Initial Screening for RVs

A total of 53 enteric samples were analyzed, with 15 samples from VAC farms (S1 and S2) and 38 samples from NON-VAC farms (S3–S6) (Table 1). In the VAC farms no genetic material of RVA or RVB was detected, while RVC was found in all the tested samples. In the NON-VAC farms, the percentage of positive samples for RVA, RVB, and RVC were 10.5% (4/38), 13.2% (5/38), and 52.6% (20/38), respectively.

**Table 1:** Collection dates and number of RVA, RVB and RVC positive samples in RVA-vaccinated (S1–S2 VAC) and non-vaccinated (S3–S6 NON-VAC) farms.

Farm ID	Date of Collection	No. of Collected Samples	No. of RVA-Positive Samples	No. of RVB-Positive Samples	No. of RVC-Positive Samples
**S1 VAC**	17 July 2023	7	0	0	7
	24 November 2023	4	0	0	4
**S2 VAC**	24 November 2023	4	0	0	4
**S3 NON-VAC**	5 July 2022	3	2	0	0
	13 September 2023	4	0	0	4
	5 December 2023	5	0	0	0
**S4 NON-VAC**	28 August 2023	3	0	0	2
	28 November 2023	7	1	0	3
**S5 NON-VAC**	5 July 2022	2	0	0	2
	5 December 2023	5	0	0	2
**S6 NON-VAC**	5 July 2022	4	1	3	3
	5 December 2023	5	0	2	4
**Total**		53	4	5	31

#### 3.1.2. Longitudinal Testing

A total of 61 ileal content samples and 28 pooled fecal samples (each pool representing two animals) from 117 piglets were analyzed, with 56 samples from S2-VAC and 33 samples from S6-NON-VAC farm (Table 2). RVA was detected in the ileum content samples from two weekly groups out of five in S2-VAC and in one weekly group out of five in S6-NON-VAC farm. No genetic material of RVA was detected in any of the tested fecal samples. RVB was found in one fecal sample from S2-VAC farm. In contrast, RVB was detected in four out of five weekly groups from S6-NON-VAC farm (48.5%; 16/33). RVC was present in all weekly groups in both studied farms and sample types.

**Figure 2:**
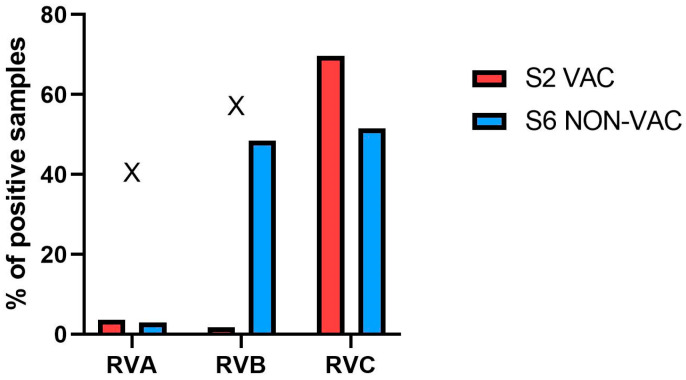
Percentage of RVA, RVB, and RVC positive samples (ileum content and fecal pools) in RVA-vaccinated (S2-VAC) and non-vaccinated (S6-NON-VAC) farms. RVC prevalence was compared using Fisher test. “X” means that statistical comparison was not performed.

The overall percentage of positive samples is presented in Figure 2. In S2-VAC farm, the percentage of positive samples for RVA, RVB, and RVC was 3.6% (2/56), 1.8% (1/56), and 69.6% (39/56), respectively. In S6-NON-VAC, RVA was also the least prevalent species (3.0%; 1/33), while the prevalence of both RVB (48.5%; 16/33) and RVC (51.5%; 17/33) was high.

The frequency of coinfections with other RV species and their possible impact on Ct values were analyzed in both sample types (ileum content and fecal pools). All RVA-positive samples were also positive for RVC (100%; 3/3). No genetic material of other RV species was detected in the RVB-positive sample from S2-VAC farm. Coinfections were present at higher rates in S6-NON-VAC farm. In this farm, RVC was present in 56.3% (9/16) of RVB-positive samples, and 52.9% (9/17) of the RVC-positive samples contained RVB. Coinfections were more frequent than single RVB infection or RVC infection in S6-NON-VAC farm; however, the difference was not statistically significant (*p* > 0.05). Moreover, Ct values from samples with a single infection and from samples positive for more than one species were similar (*p* > 0.05) (Supplementary Table S1). The median Ct value of RVB positive samples was 24.8 (22.0–33.0) and 27.6 (23.4–32.8) in coinfected and non-coinfected samples, respectively. In the case of RVC, the median Ct value was slightly higher in coinfected (18.8–29.6; median = 28.0) than in non-coinfected ones (12.0–30.6; median = 22.1). As the comparison was focused on RVB + RVC coinfections, one sample positive for RVA+RVC (without RVB) was included in the non-coinfected group. No coinfections of RVA and RVB were detected in the samples analyzed.

**Table 2:** Number of positive RVA, RVB and RVC samples and weaning performance of five consecutive weekly groups on vaccinated (S2-VAC) and non-vaccinated (S6-NON-VAC) farm.

Farm ID	Weekly Group	Sample Type	No. of Collected Samples	No. of RVA-Positive Samples	No. of RVB-Positive Samples	No. of RVC-Positive Samples	Pre-Wean Mortality [%]	No. of Weaned Piglets per Litter	Average Weaning Weight [kg]
**S2 VAC**	1	feces	2	0	0	2	11.7	15.4	5.3
		ileum content	8	1	0	5			
	2	feces	3	0	0	3	10.6	16.1	5.1
		ileum content	8	0	0	6			
	3	feces	3	0	0	3	15.6	15.2	5.4
		ileum content	8	0	0	4			
	4	feces	3	0	0	2	13.7	14.8	5.0
		ileum content	8	1	0	5			
	5	feces	5	0	1	4	15.1	14.7	5.1
		ileum content	8	0	0	5			
**S6 NON-VAC**	1	feces	1	0	1	1	13.0	12.8	5.3
		ileum content	4	1	0	2			
	2	feces	3	0	3	1	13.3	16.1	5.5
		ileum content	7	0	5	3			
	3	feces	3	0	2	2	16.6	14.3	5.5
		ileum content	2	0	2	1			
	4	feces	3	0	2	3	16.0	15.3	5.5
		ileum content	6	0	1	1			
	5	feces	2	0	0	2	15.9	14.1	5.4
		ileum content	2	0	0	1			

### 3.2. Production Data

**Figure 3:**
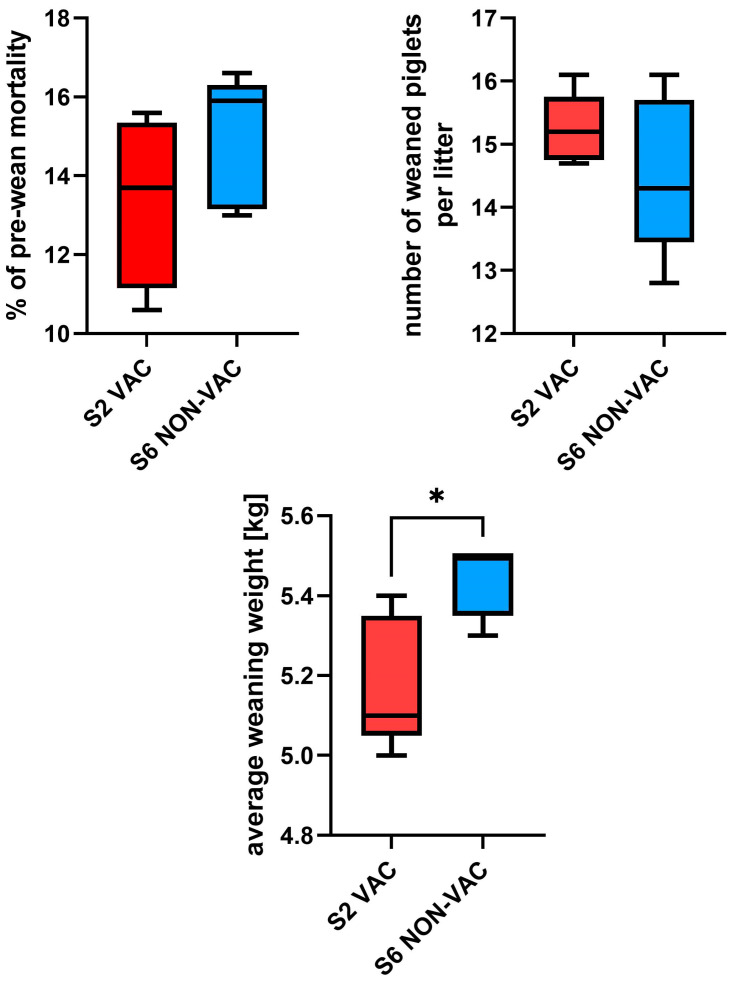
Comparison of production (pre-wean mortality, number of weaned piglets per litter, and average weaning weight) in RVA-vaccinated (S2-VAC) and non-vaccinated (S6-NON-VAC) farms. Statistical analyses were performed using Mann–Whitney test. “*” indicates *p* < 0.05.

Key performance indicators (pre-wean mortality, number of weaned piglets per litter, and average weaning weight) were obtained for five consecutive weekly groups on farm S2-VAC and S6-NON-VAC (Figure 3). The pre-wean mortality rate was slightly lower in S2-VAC (10.6–15.6; median = 13.7%) than in S6-NON-VAC (13.0–16.6; median = 15.9%). The median number of weaned piglets per litter in S2-VAC and S6-NON-VAC was 15.2 (range, 14.7–16.1) and 14.3 (range, 12.8–16.1), respectively. Weaned piglets were heavier in S6-NON-VAC (5.3–5.5 kg, median = 5.5 kg), and the difference was statistically significant (*p* < 0.05). In S2-VAC, weaning weight ranged from 5.0 to 5.4 kg (median = 5.1 kg). Production data for each weekly group are shown in Table 2.

### 3.3. Biosecurity Scoring

Biosecurity data were obtained for all six farms. Average biosecurity scores during the study were slightly higher on VAC farms (S1 and S2), with 94.5% for external biosecurity, 75.0% for internal biosecurity, and 85.0% overall. On NON-VAC farms (S3–S6), the scores were 93.8%, 67.0%, and 80.5%, respectively.

**Figure 4:**
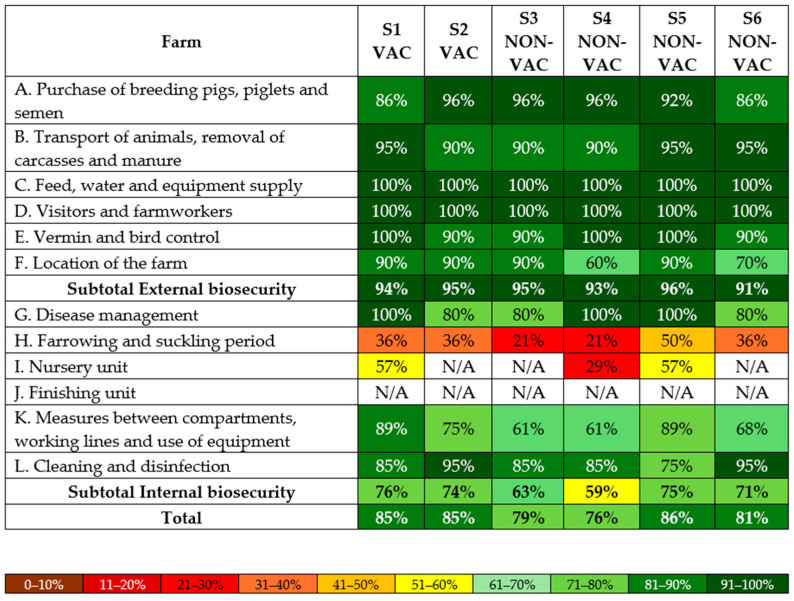
External and internal biosecurity scores for the individual RVA-vaccinated (S1–S2 VAC) and non-vaccinated (S3–S6 NON-VAC) farms.

All farms scored the lowest in the subcategory ‘Farrowing and suckling period’. In this subcategory, both VAC farms scored 36.0%, while NON-VAC farms ranged from 21.0% to 50.0%, averaging 32.0%. For ‘Measures between compartments, working lines, and use of equipment’ the average score was 82.0% (75.0% and 89.0%) for VAC, and 69.8% (range, 61.0–89.0%) for NON-VAC farms. In ‘Cleaning and disinfection’, the average score was 90.0% (85.0% and 95.0%), and 85.0% (range, 75.0–95.0%), respectively. The highest scores in internal biosecurity were obtained for ‘Disease management’, with both groups averaging 90.0%. Biosecurity scores for individual farms are shown in Figure 4.

The major noncompliance observed on all six farms was repeated cross-fostering, including mixing of the litters later than 48 h after throwing. Moreover, sows were not washed before they entered the farrowing pen, and work routines did not consistently follow the recommended sequence from younger to older pigs on any of the studied farms.

## 4. Discussion

Rotaviruses are important factors of piglet diarrhea. However, while three species (RVA, RVB, RVC) are known to cause piglet diarrhea, only RVA vaccines, autogenous and commercial, are available, and the effectiveness of the use of vaccination programs is often debatable. Moreover, there are reports indicating that immunological pressure from RVA vaccination might lead to the increase in the prevalence of RVB and RVC in swine farms [21,26]; nevertheless, data on the impact of the vaccination against RVA on the prevalence of other RV species (RVB, RVC, and RVH) remain scarce. This study aimed to determine the prevalence of RVA, RVB, and RVC in RVA-vaccinated and non-vaccinated farms and to evaluate the impact of RVA vaccination on several production parameters, in order to assess the efficiency of vaccination against RVA.

The decision to implement RVA vaccination program in the analyzed farms was made based on a prior detection of RVA in cases of pre-weaning diarrhea. RVC was also detected in the samples collected from these cases but, currently, autogenous vaccines can only be manufactured against RVA, so only this RV species could be targeted. Surprisingly, in this study RVA was only detected in few cases in the six farms regardless of their RVA vaccination status. Moreover, the longitudinal study in S2-VAC and in S6-NON-VAC, revealed slightly higher RVA detection rates in the vaccinated farm. Therefore, considering the very high RVC prevalence across the tested farms, RVC could be considered as a more important factor of piglet diarrhea, and the role of RVA was likely marginal in the period the study was performed. However, as the period of the longitudinal investigation was limited, it cannot be ruled out that RVs prevalence was impacted by different factors such as seasonal variations. In addition, as only two farms were included in the longitudinal study, the results should be interpreted with caution and may not be generalizable to other farms. Consequently, our results should be interpreted as indicative rather than exhaustive.

An increase in RVC prevalence in suckling piglets has been observed globally in recent years [27]. It has been hypothesized that this change can be related to the emergence of RVC strains with increased pathogenicity, such as American RV0104 (G3) and RV0143 (G6) viruses [27]. To our knowledge, no comparative analysis of pathogenicity of modern and historical European RVC strains has been conducted. Therefore, high rates of RVC in clinical samples from diarrheic suckling piglets in Europe such as in northeastern Spain (39.1%), Belgium (29%) and northern Italy (21.7%) might result from different unknown factors [8,17,18].

It is often assumed that RVB infections are more prevalent in older animals (over 55 days old) [14]. However, more recent studies have indicated that RVB is widespread in all age groups and cause diarrhea in piglets [12,19]. Cases of RVB infections related diarrhea outbreaks in suckling piglets, characterized by high morbidity and mortality, have been reported [20,21]. In our study, RVB was detected only in two farms—S2-VAC and S6-NON-VAC. In S2-VAC, RVB was detected only in one sample; therefore, its impact on neonatal diarrhea on this farm was likely negligible. On the other hand, in S6-NON-VAC, RVB was found in the initial screening, and in four out of five consecutive weekly groups, with an overall detection rate of 48.5% (16/33). Moreover, in April 2024 on the same farm, an outbreak of diarrhea in suckling piglets was observed, where RVB was the only viral factor detected with high virus load [22]. These data strongly suggest that in some farms RVB can be suspected as a main viral factor of piglet diarrhea.

Interestingly, there has been reported a variation in the detection rates of RVB from cases of suckling piglet diarrhea in Europe. Low prevalence in clinical samples was observed in Germany (1.6%), and northeastern Spain (9.3%), while high RVB detection rate was found in northern Italy (27%) and Switzerland (25%) [8,12,18,28]. This discrepancy might originate from different sampling periods, as the study from Germany tested samples obtained between 1999 and 2013, while samples from other studies were collected after 2016. Further possible explanations include differences in the sensitivity of the diagnostics methods, regional differences in the ways pigs were reared, as well as differences in the prevalence of pathogenic strains. RVB appears to be evolving faster than RVA and RVC when considering the number of genotypes existing in swine, with consequences difficult to predict [21]. Certainly, it could impact the sensitivity of diagnostic PCR methods. RVs genetic variability makes it difficult to develop molecular test specific to all the circulating genotype variants [19]. Further studies on RVs diagnostics are warranted to ensure reproducibility of the diagnostic techniques. In our study, two extraction kits were employed Kylt RNA/DNA Purification Kit (SAN Group Biotech Germany GmbH, Höltinghausen, Germany) and IndiSpin QIAcube HT (Indical Bioscience, Leipzig, Germany). Although both are widely used in veterinary diagnostics, potential variability in extraction efficiency cannot be fully excluded.

High prevalence of RVB in only one out of six farms belonging to a single, largely unified production system, is in strong contrast with the high prevalence of RVC in all six farms. The explanation of this discrepancy was out of scope of this work and requires further epidemiological and sequencing studies in this production system, and elsewhere in Poland.

It has been proposed that high RVA or RVC shedding is associated with increased odds of low shedding of the other species; however, high RVC shedding is associated with reduced odds of low RVB shedding [24]. In our study, RVB + RVC coinfections were more prevalent than single RVB or RVC infections in the S6-NON-VAC farm. However, we did not observe any significant impact on viral loads, inferred from Ct values for both RVC and RVB. Due to the relatively small number of analyzed samples, further studies are warranted to understand the relationships between different RV species.

The impact of diarrhea caused by RVs, and the efficiency of RVA vaccination, on the production parameters in the analyzed farms is difficult to assess since RVA was a relatively rare finding, and RVC was present in all farms. All the economic losses, if originating form RV infection, could only be attributed to RVC in herds S1–S5, and to RVC and RVB in herd S6-NON-VAC. There was no noticeable difference in clinical signs and weaning performance between single RVC infections and RVB + RVC coinfections. Surprisingly, pre-wean mortality was slightly lower (median = 13.7% and 15.9%) and the number of weaned piglets per litter was higher (median = 15.2 and 14.3) on S2-VAC farm compared to the other farm. However, the differences were not statistically significant and probably hold marginal clinical relevance, as many factors such as crushing, prematurity, and other diseases may influence the mortality. Also, the weaned piglets were significantly heavier (median = 5.5 kg and 5.1 kg) on S6-NON-VAC farm. Higher weight might result from lower average number of surviving piglets per litter, and reduced competition among littermates. Moreover, in a limited sampling period results can be greatly influenced by cofounders such as litter size at birth. These data indicate that vaccination against RVA was probably not rational, and its economic benefit might be lacking. However, it cannot be ruled out that the higher piglet survival on S2-VAC farm was an outcome of vaccination and its impact on reducing subclinical RVA pressure. Further large-scale studies with more balanced sampling are required to evaluate the true impact of preventive programs. Nevertheless, RVA vaccination have likely limited justification in herds where RVA circulation is already minimal.

RVs are highly resistant to environmental conditions, making effective disinfection difficult [5]. The severity of diarrhea and its impact on production parameters, as well as pathogens prevalence, may depend on internal biosecurity and hygiene. Rigorous biosecurity measures can reduce the number and the severity of diarrhea episodes among suckling piglets [23]. However, our preliminary observation showed higher RV prevalence on large-scale farms with high biosecurity level, than in small-scale farms with low to very low biosecurity level. It is obvious that strict disinfection might reduce the environment contamination and spread of RVs. On the other hand, it limits the sows’ exposure and might perhaps also compromise the level of maternal immunity transferred to piglets. Natural planned exposure (NPE) is a control strategy based on sows’ exposure to circulating viruses. NPE has the potential to control the disease caused by RVA as well as non-cultivable RV species such as RVC or RVB. Nevertheless, this method poses the risk of unintended spread of other pathogens [24].

There was no noticeable relationship between differences in biosecurity scores and the prevalence of RVs or the clinical status on both VAC and NON-VAC farms. However, the total obtained score may not reflect the true risk, and some of the management practices may have more impact on disease circulation than others. Interestingly, both farm types had the highest score in ‘Disease management’ and very high scores in ‘Cleaning and disinfection’. Despite such good results, or because of it, rotavirus infections were highly prevalent. This is most likely due to the cross-fostering and resilience of the virus. Further studies are warranted to better understand the relationship between biosecurity measures and prevalence of RVs.

Studies on genetic diversity are necessary for understanding of RVs epidemiology. Full-genome sequencing might explain if variation in RVs prevalence among the studied farms is a result of circulation of strains with potentially diverse pathogenic potential. The obtained results indicate the need for in-depth diagnostics of RVs presence in cases of piglet diarrhea, before the implementation of any control strategy.

## Data Availability

The data presented in this study are available on request.

## Supplementary Materials

The following supporting information can be downloaded at https://www.mdpi.com/article/10.3390/pathogens14101055/s1, Table S1: Ct values of RVs-positive samples from S6-NON-VAC farm.

## Abbreviations

The following abbreviations are used in this manuscript:

RVs	Rotaviruses
RVA	Rotavirus A
RVB	Rotavirus B
RVC	Rotavirus C
RVD	Rotavirus D
RVF	Rotavirus F
RVJ	Rotavirus J
RVK	Rotavirus K
RVL	Rotavirus L
PEDV	Porcine epidemic diarrhea virus
TGEV	Transmissible gastroenteritis virus
RVE	Rotavirus E
RVH	Rotavirus H
VAC	Vaccinated
NON-VAC	Non-vaccinated
SDCoV	Swine delta coronavirus
NPE	Natural planned exposure
