# Supplementary material for: Prevalence of Rotavirus in Diarrheic Piglets on RVA-Vaccinated and Non-Vaccinated Farms
Source: Pathogens. 2025 Oct 18;14(10):1055. doi: 10.3390/pathogens14101055 (PMC12567190; doi:10.3390/pathogens14101055)
Supplement: Supplementary file 1 [file pathogens-14-01055-s001.zip › pathogens-3896808-supplementary.pdf]

Supplementary Table S1. Ct values of RVs-positive samples from S6-NON-VAC farm. Ct ≤ 37 was considered positive.

		Ct value		
**Weekly group**	**Sample type**	**RVA**	**RVB**	**RVC**
1	feces	-	21.97	28.20
	ileum content	-	-	12.06
	ileum content	34.05	-	30.57
2	ileum content	-	24.63	-
	ileum content	-	25.18	27.59
	ileum content	-	31.73	27.95
	ileum content	-	33.01	21.94
	ileum content	-	32.73	-
	feces	-	24.69	-
	feces	-	24.44	28.46
	feces	-	27.59	-
3	ileum content	-	24.82	28.79
	ileum content	-	32.29	-
	feces	-	-	28.24
	feces	-	22.93	26.44
	feces	-	23.44	-
4	ileum content	-	32.77	-
	ileum content	-	-	23.60
	feces	-	24.15	29.63
	feces	-	-	27.58
	feces	-	31.53	18.80
5	ileum content	-	-	13.56
	feces	-	-	20.57
	feces	-	-	18.29
**Ct values [min-max; median]**			21.97-33.01; 25.00	12.06-30.57; 27.58
